# Brensocatib—Another Therapeutic “Window of Opportunity” for Patients with Bronchiectasis

**DOI:** 10.3390/jcm15031257

**Published:** 2026-02-04

**Authors:** Florin-Dumitru Mihălțan, Ruxandra Ulmeanu, Ancuța-Alina Constantin

**Affiliations:** 1 Department of Cardio-Thoracic Pathology, “Carol Davila” University of Medicine and Pharmacy, 050474 Bucharest, Romania; florin.mihaltan@umfcd.ro; 2 Institute of Pneumology “Marius Nasta”, 050159 Bucharest, Romania; 3 Faculty of Medicine and Pharmacy, Doctoral School of Biomedical Sciences, University of Oradea, 410073 Oradea, Romania

**Keywords:** bronchiectasis, brensocatib, exacerbation, dipeptidyl peptidase-1 (DPP-1)

## Abstract

**Introduction:** Bronchiectasis is a chronic, heterogeneous airway disease characterised by irreversible bronchial dilatation, recurrent infections, and persistent inflammation, leading to progressive lung damage, frequent exacerbations, and impaired quality of life. Neutrophil-driven inflammation, largely mediated by excessive activity of neutrophil serine proteases such as neutrophil elastase, represents a central pathogenic mechanism and an important therapeutic target. **Methods**: Brensocatib, a first-in-class, selective, and reversible inhibitor of dipeptidyl peptidase-1 (DPP-1), prevents the activation of neutrophil serine proteases during neutrophil maturation in the bone marrow. By reducing downstream protease activity, brensocatib modulates aberrant neutrophilic inflammation without broadly suppressing immune function. **Results:** Clinical studies, including the Phase-2 WILLOW trial and the Phase-3 ASPEN trial, have demonstrated that brensocatib significantly reduces exacerbation frequency, prolongs time to first exacerbation, and lowers sputum neutrophil protease activity, with a favourable safety profile. Importantly, these benefits were observed across multiple patient subgroups and in addition to standard-of-care therapies. **Conclusions:** As the first FDA-approved (12 August 2025) mechanism-based therapy for non–cystic fibrosis bronchiectasis, brensocatib represents a paradigm shift toward targeted, precision treatment of neutrophil-mediated airway disease. Its clinical efficacy, biomarker-driven rationale, and potential to reduce antibiotic dependence highlight brensocatib as a cornerstone therapy in bronchiectasis management and a promising strategy for other neutrophil-driven inflammatory conditions.

## 1. Introduction

Bronchiectasis is a progressive lung disease resulting from a “vicious cycle” of recurrent bacterial infections and a dysregulated inflammatory response. It is characterised by irreversible airway dilatation, chronic inflammation, and impaired mucociliary clearance. At the same time, bronchiectasis remains a highly heterogeneous condition arising from a wide range of aetiological factors associated with immune dysregulation, spanning immunodeficiency states to immune excess with inflammatory hyperactivation. These mechanisms may be triggered by severe viral, bacterial, or fungal infections, as well as autoimmune disorders.

The establishment of new international registries, multicentre studies, and advances in bronchiectasis endotyping have opened the way to novel therapeutic strategies. Current bronchiectasis therapies face limitations due to the disease’s complexity and heterogeneity, including a lack of specifically licenced drugs; reliance on low-evidence treatments such as inhaled corticosteroids (ICS), which are often ineffective outside asthma/COPD overlap; insufficient data supporting promising options like triple inhalers (LABA/LAMA/ICS); and inconsistent adherence to essential airway clearance techniques (ACTs). These limitations highlight the need for stratified, evidence-based approaches. Despite increased research, no drug is universally approved by regulatory bodies specifically for bronchiectasis, in contrast to other lung diseases, as noted in this review [1,2]. However, previous efforts to suppress neutrophilic inflammation have been largely unsuccessful. These approaches either excessively inhibited neutrophil migration, potentially increasing susceptibility to infection, or failed to adequately block neutrophil elastase (NE) activity [3,4].

More recently, the development of drugs targeting DPP-1—the enzyme responsible for activating NE proteases during their maturation in the bone marrow—has opened a new therapeutic avenue for controlling inflammation in bronchiectasis [5,6].

## 2. General Aspects Concerning Bronchiectasis

The incidence and prevalence of bronchiectasis are increasing worldwide, particularly among the elderly population. Prevalence estimates reach up to 566 cases per 100,000 individuals in the United Kingdom [7], with an overall mortality rate of 20.4% reported over a 5-year follow-up period [8]. The coexistence of chronic obstructive pulmonary disease (COPD) and bronchiectasis shows wide geographic variability, with reported prevalence rates ranging from 30.1% to 52% in the United States, 39% in Germany, 23.2% in Italy, 19.3% in South Korea, and 8.3% in Singapore [9]. Considering these caveats, existing data suggest that the prevalence of bronchiectasis may be lower in continental Europe (53–362 per 100,000 individuals) [10] than in Asia (76–1249 per 100,000 individuals) [11]. Different continental European cohorts have reported mortality rates ranging from 16% to 24.8%, with follow-up periods of 4.0–5.18 years [12]. Greater heterogeneity has been reported in Asia and Australia, where mortality rates range from 2.3% to 21% with follow-up periods of 1–10 years [13,14].

Other significant comorbid conditions frequently associated with bronchiectasis are chronic rhinosinusitis, reported in 32–80% of patients [9,15], while asthma prevalence varies geographically, affecting 28.3–29% of patients in the United States, 31% in the United Kingdom and Europe, 16% in Italy, 17.2–22% in South Korea, 22.1% in India, 5.4% in China, and 3.6% in Singapore [15]. Approximately 46% of patients with bronchiectasis have comorbid asthma; however, comorbidity with severe asthma appears to be limited, as shown in 3.3% of bronchiectasis cases [16].

Bronchiectasis also carries a substantial economic burden. Total annual healthcare costs per adult patient may reach up to USD 82,000, driven predominantly by disease exacerbations and hospitalisations [17]. Data from international registries focusing on aetiology-associated phenotypes indicate that 29–41% of bronchiectasis cases are classified as idiopathic [18]. Post-infective bronchiectasis accounts for approximately 20–27% of cases [19]. In addition, 13% of patients with bronchiectasis were diagnosed with nontuberculous mycobacterial pulmonary disease (NTM-PD) in a Southern European bronchiectasis registry [20].

Regarding disease phenotyping, the most well-characterised bronchiectasis phenotype is chronic bronchial infection with *Pseudomonas aeruginosa*. The combination of *P. aeruginosa* infection and frequent exacerbations is associated with poorer clinical outcomes [21]. The frequent exacerbator phenotype is independently associated with increased hospitalisation rates and higher 5-year mortality [22]. Other phenotypes described in the literature include the high symptom burden phenotype [23] and the chronic airway disease overlap phenotype, encompassing coexisting COPD or asthma, which affects approximately 29–60% of patients [24].

## 3. Phenotypes, Triggers, Quality of Life, and Patient Concerns

The clinical manifestations of bronchiectasis commonly include daily productive cough, dyspnoea, fatigue, and increased susceptibility to recurrent respiratory tract infections [25,26]. Repeated exacerbations are associated with accelerated lung function decline, increased mortality, and a significant deterioration in quality of life [27,28]. Data from the European Multicentre Bronchiectasis Audit and Research Collaboration (EMBARC) registry indicate that more than one-third of patients across all regions can be classified as frequent exacerbators, defined as experiencing two exacerbations or one hospitalisation per year [29].

The heterogeneity of bronchiectasis signs and symptoms reflects differences in underlying aetiology, abrupt changes in the lung microbiome and airway homeostasis, and the presence and activity of comorbidities [30]. Phenotyping plays a crucial role in assessing disease burden and patient-reported quality of life. The frequent exacerbator phenotype is characterised by factors such as chronic infection with *Haemophilus influenzae* or *Pseudomonas aeruginosa*, reduced forced expiratory volume in one second (FEV_1_), greater radiological disease severity, coexisting chronic obstructive pulmonary disease (COPD), and increased symptom burden. All of these factors are independent risk predictors for future exacerbations in bronchiectasis. Patients who are highly symptomatic derive a similar benefit from long-term macrolide treatment as patients with a high baseline exacerbation frequency [22,31].

The neutrophilic phenotype is the most common and well-characterised inflammatory pattern in bronchiectasis. Neutrophils are the dominant inflammatory cells both in the stable state and during exacerbations. Neutrophilic accumulation results from the host’s response to microbial attack, followed by the release of pro-inflammatory mediators such as interleukin (IL)-1β, IL-8, IL-17, leukotriene B4, and tumour necrosis factor-α (TNF-α), which drive immune-cell accumulation and degranulation [32].

The eosinophilic phenotype, estimated to account for approximately 20% of cases, exhibits an eosinophilic inflammatory profile. This phenotype is defined as ≥3% eosinophils in sputum or ≥300 cells/μL in peripheral blood, in the absence of comorbidities that independently increase blood eosinophil counts [33].

In addition, a distinct T2-high bronchiectasis endotype has been identified, separate from asthma-related phenotypes. This inflammatory endotype is characterised by specific airway microbiome profiles and elevated blood eosinophil counts (>300 cells·μL^−1^) and is associated with a shortened time to first exacerbation following antibiotic treatment for *P. aeruginosa* infection [33].

Mixed neutrophilic–eosinophilic phenotype bronchiectasis is defined by the co-expression of neutrophilic markers (e.g., neutrophil elastase [NE], IL-8, TNF-α) and eosinophilic markers (IL-5, IL-13, FeNO) [34].

The paucigranulocytic phenotype, another expression of bronchiectasis, is characterised by <60% neutrophils and <3% eosinophils in sputum, with no clear dominance of other inflammatory cell types [35]. This phenotype is associated with reduced levels of inflammatory mediators compared with neutrophilic or eosinophilic phenotypes; IL-8 and IL-13 levels are significantly lower, as are FeNO levels [36].

Triggers of bronchiectasis exacerbations can be broadly categorised into exogenous and endogenous factors. Exogenous triggers include acute and chronic bacterial infections—most commonly involving *P. aeruginosa*, *H. influenzae*, *Moraxella catarrhalis*, *Enterobacterales*, *Staphylococcus aureus*, and *Streptococcus pneumoniae*—as well as viral infections such as rhinovirus, influenza A and B, and respiratory syncytial virus (RSV). Fungal infections, particularly aspergillosis, also contribute, often in association with microbiome dysregulation or “dysbiosis,” which may be further altered by antibiotic exposure [37]. Environmental factors, including air pollution, along with nutritional status, have also been implicated as important modulators of exacerbation risk [38,39]. Based on inflammatory and microbiological characteristics, exacerbations have been proposed to occur in bacterial, viral, bacterial–viral co-infection, eosinophilic, and unidentified forms, each associated with distinct inflammatory and microbiome profiles [40].

Endogenous factors such as inflammatory endotypes also play a significant role in exacerbation pathogenesis. Neutrophilic inflammation, driven by NE—a protease released from azurophilic granules of activated neutrophils—leads to increased mucin secretion and impaired mucociliary clearance. Additionally, the formation of neutrophil extracellular traps, consisting of DNA webs and granular contents released during dysregulated neutrophilic responses, contributes to airway damage and persistent inflammation [41,42]. Endogenous factors associated with increased exacerbation risk include reduced microbiome diversity and *P. aeruginosa* infection, gastroesophageal reflux disease (GERD), chronic rhinosinusitis, and poor adherence to treatment for both bronchiectasis and associated comorbid conditions [38,39].

## 4. Immunopathology of Bronchiectasis: The Central Role of Neutrophils

Although eosinophilic inflammation and systemic inflammation are increasingly recognised in bronchiectasis, the disease remains predominantly neutrophil-driven. Neutrophilic inflammation is characterised by excessive protease activity, impaired bacterial clearance, and a high burden of exacerbations. NE accounts for approximately 80% of total proteolytic activity in the airways and is considered one of the most destructive protein-degrading enzymes in the human body [43].

Neutrophil recruitment to the airways is mediated by pro-inflammatory cytokines and chemokines, including CXCL8, interleukin (IL)-1β, tumour necrosis factor-α (TNF-α), as well as neutrophil serine proteases such as NE. All of these mediators are markedly increased in sputum and bronchoalveolar lavage fluid from patients with bronchiectasis [44,45]. Neutrophils play a central role in innate immunity through multiple effector mechanisms, including phagocytosis, degranulation, and the formation of neutrophil extracellular traps (NETs).

While NET formation contributes to pathogen elimination, excessive or dysregulated NET release promotes tissue damage and sustained inflammation. NETs contain high concentrations of NE, which contributes to airway tissue injury, impaired bacterial clearance, and mucus hypersecretion. Importantly, sputum NE levels have been shown to correlate strongly with exacerbation risk, lung function decline, and mortality in bronchiectasis [41].

Much of the neutrophil dysfunction observed in bronchiectasis is consistent with immunometabolic reprogramming [46]. Targeting neutrophils by reducing their numbers has been associated with an increased frequency of infections. Consequently, newer approaches focus on targeting metabolism, which could theoretically reverse neutrophil dysfunction and dysregulated inflammation. In models of pulmonary disease, 5′-adenosine monophosphate (AMP)–activated protein kinase (AMPK) activation has been shown to reverse phagocytic dysfunction and NET formation. AMPK modulates multiple metabolic pathways, including glycolysis, which is critical for energy generation in neutrophils. AMPK activators can reverse metabolic reprogramming and are already in clinical use and/or development. Therefore, some authors propose a novel immunomodulatory approach rather than a purely anti-inflammatory approach to enhance bacterial clearance and reduce bronchiectasis severity [47].

Despite the presence of large numbers of neutrophils in the airways, bacterial infection often persists in bronchiectasis, indicating impaired neutrophil function. Evidence suggests that neutrophils become functionally disabled through multiple mechanisms, including cleavage of phagocytic receptors by NE and inhibition of phagocytosis by neutrophil-derived peptides [48]. Targeting neutrophils faces several limitations, including their essential role in host defence; the activation of compensatory pathways (e.g., other immune cells) when neutrophil function is blocked; difficulty distinguishing harmful from protective functions due to neutrophil plasticity; and challenges in therapy delivery, which can lead to off-target effects or resistance, particularly in the context of NETs.

Dipeptidyl Peptidase-1, also known as Cathepsin C, is a key enzyme targeted for treating bronchiectasis, with DPP-1 inhibitors reducing the damaging effects of neutrophil enzymes and improving lung function, quality of life, and exacerbation rates. DPP-1, a cysteine protease essential for the activation of neutrophil serine proteases such as NE during neutrophil maturation, has also emerged as a promising therapeutic target. Pharmacological inhibition of DPP-1 with agents such as brensocatib reduces neutrophil serine protease activity and attenuates neutrophil-mediated inflammation [23]. Accumulating evidence indicates that DPP-1 inhibition can modulate the aberrant host immune response, reduce mucus production, and potentially slow disease progression in bronchiectasis [49]. Moreover, inhibition of DPP-1 has been shown to protect against inflammatory and immune-mediated diseases by reducing neutrophil infiltration at sites of inflammation, inactivating neutrophil serine proteases, and decreasing NET formation [50].

Beyond neutrophil-driven inflammation, several additional mechanisms contribute to the subversion of innate immune mechanisms in bronchiectasis. These include bacterial strategies such as biofilm formation, reduced bacterial motility, and downregulation of virulence factors, which collectively promote immune evasion and chronic airway infection [51]. Natural killer (NK) cells, acting as innate lymphoid cells, are present in the lung as both circulating and tissue-resident populations. They play a key role in airway immune surveillance and participate in immune crosstalk relevant to chronic airway diseases, including COPD. Emerging evidence suggests that NK cells may also contribute to the development and progression of bronchiectasis. In this context, airway-resident NK cells may exert dual effects on neutrophils, promoting neutrophil survival following stimulation by pro-inflammatory cytokines, while also facilitating neutrophil apoptosis under specific inflammatory conditions [52,53].

Neutrophil-derived inflammatory mediators, including IL-8 and TNF, can stimulate airway epithelial and goblet cells to increased mucus secretion [54]. Neutrophils further amplify mucus-associated inflammation through interactions with other immune cell populations [55]. In addition, neutrophils directly affect airway structural integrity by releasing proteolytic enzymes and other bioactive substances, contributing to tissue damage and airway remodelling [56]. Neutrophilic inflammation persists even in clinically stable bronchiectasis, including in the absence of active infection. In the stable state of bronchiectasis, peripheral blood neutrophils are reprogrammed and exhibit prolonged survival. This impairs their ability to phagocytose and kill bacteria, thereby perpetuating the vicious circle of bronchiectasis [57].

During activation, neutrophils generate substantial amounts of reactive oxygen species (ROS), which are essential for microbial killing but also induce oxidative damage to host tissues [58]. Furthermore, reprogrammed or dysregulated neutrophils preferentially accumulate at sites of inflammation and release high levels of pro-inflammatory mediators, thereby exacerbating local airway inflammation and potentially driving systemic inflammatory responses [59].

Brensocatib does not appear to cause clinically significant impairment of immune function. This observation is supported by evidence from Papillon–Lefèvre syndrome, in which loss-of-function mutations in DPP-1 lead to markedly reduced neutrophil serine protease activity without resulting in overt immunodeficiency. Consistent with this, follow-up analyses from clinical studies have confirmed broad inhibition of sputum neutrophil serine proteases—including NE, proteinase 3 (PR3), and cathepsin G—following brensocatib treatment [60].

As an indirect inhibitor of NE activation, brensocatib does not restore neutrophil differentiation, indicating that its therapeutic effects are mediated through modulation of neutrophil effector function rather than correction of neutrophil developmental pathways [61]. Importantly, brensocatib does not induce widespread, nonspecific suppression of neutrophil secretory products, as evidenced by the absence of significant changes in sputum myeloperoxidase (MPO) levels between treatment and placebo groups [62].

Studies evaluating the downstream effects of DPP-1 inhibition beyond neutrophil serine proteases have provided additional insights. Analyses assessing secretory leukoprotease inhibitor (SLPI), α-defensin-3, MPO, and the mucins MUC5AC and MUC5B showed that treatment with brensocatib was associated with reduced sputum NE activity. Given the downstream effects of NE inhibition, including reduced degradation of SLPI, the observed increase in sputum SLPI levels among treated patients was anticipated [63]. Additionally, the increase in α-defensin-3 reported by Johnson and colleagues appears to result from reduced proteolytic degradation by NE and related proteases rather than from direct upregulation of peptide synthesis. α-Defensin-3 (human neutrophil peptide-3) is one of several antimicrobial peptides secreted primarily by neutrophils and plays a role in host defence and immune regulation [64]. In contrast, MUC5B levels were not significantly altered by brensocatib treatment, suggesting that DPP-1 inhibition does not broadly affect mucin expression.

Collectively, these findings highlight neutrophils as central pathogenic drivers in bronchiectasis and underscore their importance as therapeutic targets. Recent advances in the pharmacological management of bronchiectasis have demonstrated promising clinical potential through targeted inhibition of neutrophil-derived proteases, as supported by emerging clinical trial data.

## 5. Brensocatib for the Treatment of Inflammatory Pathways in Bronchiectasis

Cathepsin C, also known as DPP-1, is a cysteinyl protease belonging to the lysosomal papain family and plays a critical role in intracellular protein processing [65]. During neutrophil maturation, DPP-1 activates neutrophil serine proteases—including NE, proteinase 3 (PR3), and cathepsin G—by cleaving N-terminal dipeptides from their inactive precursors. Inhibition of DPP-1 therefore prevents activation of these proteases and has been shown to attenuate neutrophilic inflammation, modulate aberrant host immune responses, reduce mucus production, and potentially slow bronchiectasis progression [49].

Brensocatib is a first-in-class, selective, and reversible oral inhibitor of DPP-1, representing a novel therapeutic approach for inflammatory aspects of bronchiectasis. It demonstrates nanomolar-level inhibitory activity against human DPP-1, with high selectivity over related proteases such as DPP-4 and DPP-8/9 [66]. Despite recent advances in bronchiectasis management, there remains a substantial unmet need for targeted anti-inflammatory therapies. Stratifying patients with elevated neutrophilic inflammation—based on biomarkers such as NE activity or NETs—may enable precision treatment with agents such as brensocatib, which has a robust preclinical and clinical development background.

In preclinical studies, brensocatib exhibited high selectivity for DPP-1 over other cathepsins, with half-maximal inhibitory concentration (IC_50_) values exceeding 20 μM for related human recombinant cathepsins [67]. In neutrophil progenitor cells, brensocatib completely inhibited activation of NE, PR3, and CatG in a concentration-dependent manner [66]. Pharmacokinetic studies demonstrated dose-dependent and supra-proportional increases in plasma concentrations, with peak levels observed 0.5–1.5 h after dosing, followed by a multiphasic decline. The terminal half-life ranged from 20 to 26 h after single dosing and increased to 26–34 h with repeated administration [66]. Importantly, in vitro data suggest that treatment with reversible DPP-1 inhibitors such as brensocatib is unlikely to cause clinically relevant impairment of cytotoxic T lymphocyte (CTL) or natural killer cell–mediated immune responses [68], supporting its favourable safety and immunological profile.

The recent WILLOW study has provided compelling evidence for the central role of neutrophil serine proteases in the pathophysiology of bronchiectasis. WILLOW was a Phase-2, randomised, placebo-controlled clinical trial evaluating brensocatib (INS1007). Brensocatib markedly reduces neutrophil serine protease activity in sputum samples from patients with bronchiectasis and significantly prolongs the time to first exacerbation compared with placebo at two dose levels. Additionally, it significantly reduced the time to first exacerbation and lowered exacerbation risk by approximately 40% compared with placebo; it may also help lessen reliance on antibiotics and potentially curb the rise in antimicrobial resistance [46,53,62].

In patients with non–cystic fibrosis bronchiectasis treated with oral brensocatib at doses of 10 mg and 25 mg over a 24-week period, the study demonstrated clinically meaningful reductions in exacerbation frequency and hospitalisation rates. Specifically, the 10 mg dose was associated with a 42% reduction in the risk of exacerbations and hospitalisations, while the 25 mg dose resulted in a 38% reduction [69]. These findings highlight the potential of brensocatib as an alternative anti-inflammatory strategy that may reduce reliance on long-term antibiotic therapy and thereby help mitigate the emergence of antimicrobial resistance. Mechanistic validation of these clinical outcomes was further provided by the WILLOW-89 study, which confirmed the potent inhibition of neutrophil serine protease activity—including NE and PR3—by brensocatib. This inhibition effectively restored the protease–antiprotease balance and attenuated neutrophil-driven airway inflammation and parenchymal tissue damage, key pathological features of bronchiectasis [60].

More recently, the Phase-3 ASPEN trial confirmed the efficacy of brensocatib in reducing exacerbation frequency in patients with bronchiectasis, corroborating the benefits observed in earlier Phase-2 studies [62,70]. ASPEN is the largest clinical trial programme in bronchiectasis to date, enrolling over 1600 patients across 35 countries. Sputum samples were sent to a central laboratory for culture to identify patients with *P. aeruginosa* for stratification purposes, and patients underwent lung function testing, including spirometry (pre- and post-bronchodilator FEV_1_ and percent predicted FEV_1_ [ppFEV_1_]), forced vital capacity (FVC), forced expiratory flow between 25% and 75% of vital capacity, and peak expiratory flow rate. Designed to evaluate the impact of two brensocatib doses [10 mg and 25 mg] on exacerbation rate over a 52-week treatment period versus placebo, the study demonstrated that lung function was lower among *P. aeruginosa*–positive patients (mean ± SD post-bronchodilator ppFEV_1_ 64.7 ± 21.0% vs. 77.9 ± 23.4%). In addition, the ASPEN-10 investigation provided comprehensive insights into protocol design, baseline demographics, and clinical characteristics of non–cystic fibrosis bronchiectasis populations treated with brensocatib, thereby establishing a robust framework for its therapeutic application [71].

Another important conclusion of the study was that patients with *P. aeruginosa* had greater use of long-term antibiotics than those without *P. aeruginosa*, including higher overall long-term macrolide use (21.5% vs. 14.0%) and more frequent use of ICS (63.5% vs. 53.9%). Among patients with bronchiectasis, once-daily treatment with brensocatib (10 mg or 25 mg) resulted in a lower annualised rate of pulmonary exacerbations than placebo, and the decline in FEV_1_ was less with the 25-mg dose of brensocatib than with placebo. As a reversible inhibitor of cathepsin C that blocks activation of neutrophil serine proteases during neutrophil maturation, brensocatib appears to exert broad anti-inflammatory effects extending beyond protease inhibition alone. Emerging evidence suggests that these effects translate into significant clinical benefits, including sustained reductions in pulmonary exacerbation rates and attenuation of FEV_1_ decline [60,70,72,73]. Notably, post hoc analyses have consistently demonstrated that brensocatib reduces sputum activity of neutrophil proteases, including NE, reinforcing its biological activity and mechanistic rationale in bronchiectasis treatment [60].

### Critical Points Regarding Brensocatib

Despite these promising results, important questions remain regarding the long-term impact of brensocatib on morbidity, health-related quality of life, healthcare utilisation, and overall treatment costs. In addition, more robust evidence is still needed regarding its influence on mortality. Notably, inflammatory biomarkers showing the greatest response to treatment were those most closely correlated with baseline NE activity. However, these associations do not definitively establish causality, and further mechanistic studies are required to clarify whether reduced NE activity alone accounts for the observed biomarker changes.

Although clinical trials of DPP-1 inhibitors in bronchiectasis have yielded encouraging results, several limitations remain. These include relatively small sample sizes, regional variability among study populations, short treatment durations limiting assessment of long-term efficacy and safety, and uncertainties regarding cost, regulatory approval, accessibility, and affordability across different healthcare systems.

Brensocatib does not appear to cause substantial immune function suppression, as demonstrated by observations in Papillon–Lefèvre syndrome, in which DPP-1 mutations lead to reduced neutrophil serine protease activity without overt immunodeficiency [60]. Regarding anti-inflammatory effects and safety, the most frequently reported adverse reactions include headache (9.2%), hyperkeratosis (5.9%), dermatitis (4.2%), rash (4.1%), upper respiratory tract infections (3.9%), and dry skin (3.0%) [72]. Rare adverse effects included hair loss and non-melanoma skin cancers.

DPP-1 inhibitors are also being investigated for use in patients with bronchiectasis–COPD overlap syndrome (BCOS) [73]. Although brensocatib has demonstrated efficacy in bronchiectasis, the exacerbation-prone phenotype in COPD alone has been insufficiently studied [74]. Nevertheless, the drug appears particularly valuable for treating disease phenotypes characterised by neutrophilic inflammation and frequent exacerbations, especially in patients with bronchiectasis overlap [70].

## 6. Other Potential Indications, Dosing Considerations, and Safety Profile of Brensocatib

Eligible participants in the majority of clinical studies were between 12 and 85 years of age and had a clinical history consistent with symptomatic non–cystic fibrosis bronchiectasis (NCFB), confirmed by computed tomographic (CT) scan (e.g., cough, recurrent respiratory infections). Adult participants were required to have a history of at least two pulmonary exacerbations in the 12 months prior to screening and a body mass index (BMI) ≥18.5 kg/m^2^ at screening. Key exclusion criteria included a primary diagnosis of chronic obstructive pulmonary disease (COPD) or asthma, bronchiectasis due to cystic fibrosis, current smoking, known or suspected immunodeficiency disorders, and current treatment for nontuberculous mycobacterial (NTM) lung infection [75].

Clinical trial data indicate that the efficacy of brensocatib is consistent across multiple pre-specified subgroups, suggesting that a broad patient population may benefit from treatment. Importantly, brensocatib was administered in addition to standard-of-care therapies, including macrolides, inhaled antibiotics, inhaled corticosteroids, and other commonly used pharmacological agents, with clinical benefit observed regardless of background treatment [70].

Determining the optimal brensocatib dose at the individual patient level remains challenging [76]. Evidence suggests a trend favouring the 25 mg dose in some patients; Once-daily treatment with brensocatib (10 mg or 25 mg) led to a lower annualised rate of pulmonary exacerbations than placebo, and the decline in FEV_1_ was less with the 25 mg dose of brensocatib than with placebo [70]. However, many individuals achieve adequate clinical responses with the 10 mg dose. It is likely that a subset of patients may benefit from dose escalation to 25 mg, while others may not derive therapeutic benefit at either dose. The potential for additive or synergistic effects when brensocatib is combined with other therapies, such as N-acetylcysteine or inhaled antibiotics, warrants further investigation [76].

From a pharmacokinetic perspective, brensocatib can be administered with or without food. However, caution may be required in patients receiving concomitant cytochrome P450 inhibitors, as these agents may increase systemic drug exposure [77]. Regarding ethnic variability, a small pharmacokinetic study reported no significant differences in brensocatib metabolism between Japanese and White adult populations [72].

The most frequently reported adverse events associated with brensocatib include skin rash, hyperkeratosis, dry skin, changes in gingival and periodontal health, upper respiratory tract infections, headache, elevated blood pressure, and mild increases in liver enzyme levels. Rare adverse events have included alopecia and non-melanoma skin cancers. Live attenuated vaccines are contraindicated during treatment with brensocatib [78]. Notably, the drug does not contribute to the development of antimicrobial resistance [79]. Among treatment-related adverse events, COVID-19, cough, headache, nasopharyngitis, dyspnoea, and diarrhoea were most commonly reported in clinical trials [62].

Further studies are required to evaluate the efficacy and safety of brensocatib in pregnant and breastfeeding individuals, as well as in paediatric populations, particularly children under 12 years of age. Brensocatib has recently become the first approved treatment for non–cystic fibrosis bronchiectasis in adults and adolescents [80]. A boxed warning highlights the risks of hyperkeratosis and periodontal disease. In patients with renal impairment, single-dose studies have demonstrated good tolerability, suggesting that dose adjustment is not required in this population [81]. Regarding drug interactions, it is unknown whether administration of live attenuated vaccines during brensocatib treatment affects vaccine safety or efficacy. Data are unavailable on use during pregnancy to assess the risk of major birth defects, miscarriage, or other adverse maternal or fetal outcomes (Product Information. Brinsupri (brensocatib) Insmed Incorporated). Dosage for adolescents and adults is 10 mg or 25 mg orally once daily. Based on September and October 2025 reports from the Institute for Clinical and Economic Review (ICER) [75], brensocatib is considered a clinically effective treatment for non–cystic fibrosis bronchiectasis (NCFB); however, it is currently regarded as having low long-term value for money at its announced U.S. launch price ($88,000 per year). ICER concluded that, at this price, brensocatib does not meet commonly used cost-effectiveness thresholds.

### Comparison with Other Drugs

BI 1291583 (verducatib) is a cathepsin C (CatC) inhibitor expected to restore the protease–antiprotease balance in the lungs of patients with chronic airway inflammatory diseases such as bronchiectasis. It has demonstrated a superior in vivo profile for the treatment of bronchiectasis [77].

Regarding macrolides, brensocatib demonstrated efficacy in patients regardless of maintenance macrolide use (without vs. with). Annualised exacerbation rates were lower with brensocatib (10 mg: 0.97/1.21; 25 mg: 0.98/1.21) compared with placebo (1.23/1.54), and a greater proportion of patients remained exacerbation-free across subgroups. Brensocatib 25 mg also reduced FEV_1_ decline in both subgroups. In addition, the 25-mg dose reduced both FEV_1_ and FVC decline and numerically improved QOL-B respiratory symptom score at week 52 compared with placebo, regardless of baseline macrolide use [82].

With its anti-inflammatory effects, brensocatib represents a potential therapeutic opportunity for overlap syndromes involving COPD and asthma. Additional key areas for future research include evaluation of combination strategies with other anti-inflammatory therapies (such as macrolides), assessment of efficacy across distinct bronchiectasis endotypes, optimisation of dosing regimens to balance efficacy and safety, and exploration of the potential role of early intervention to slow disease progression [43].

## 7. Conclusions

Several important questions remain to be addressed in bronchiectasis. As observed in other chronic airway diseases, impaired NK cell function correlates with disease severity and exerts a modulatory effect on T-cell–mediated immune responses. In bronchiectasis, NK cells have been implicated in the regulation of neutrophil survival, further highlighting their potential role in disease pathogenesis [53].

The investigation of molecular endotypes—classifying disease based on underlying pathobiological mechanisms and treatment responsiveness—offers a more comprehensive framework for understanding bronchiectasis heterogeneity. Such an approach may enable clearer identification of patient subgroups most likely to benefit from targeted therapies, including brensocatib, across distinct endo- and exophenotypes [23].

A deeper understanding of how neutrophils drive disease initiation and progression, together with strategies aimed at selectively targeting specific neutrophil functions, is essential for advancing personalised and precision-based management of bronchiectasis. These efforts are expected to translate into improved clinical outcomes and enhanced quality of life for affected patients. In this context, brensocatib represents a significant step toward personalised therapy in a complex and heterogeneous disease such as bronchiectasis [83].

DPP-1 inhibitors, including brensocatib, have demonstrated the ability to reduce exacerbation frequency, prolong time to first exacerbation, and improve respiratory symptoms without compromising safety [84]. As a pioneering, mechanism-based therapy, brensocatib remains a cornerstone in bronchiectasis management and holds broader therapeutic implications for other neutrophil-driven conditions, including chronic obstructive pulmonary disease, asthma, chronic rhinosinusitis, alpha-1 antitrypsin deficiency, acute respiratory distress syndrome, autoimmune diseases, and cystic fibrosis [70,85].

With brensocatib representing the first approved mechanism-specific therapy for bronchiectasis, DPP-1 inhibition marks a paradigm shift in the treatment of neutrophil-mediated respiratory diseases and opens new avenues for targeted anti-inflammatory intervention [86].

## Data Availability

No new data were created or analyzed in this study.
